# From fragmentation to integration: digital health as a catalyst for universal health coverage in Somalia

**DOI:** 10.3389/frhs.2026.1929703

**Published:** 2026-10-13

**Authors:** Ilyas Abdullahi khalif, Aniso Mohamed Abdi, Yusuf Abdullahi Hubow, Abdirahman Mohamed Adan, Maryama Ahmed Ali, Ahmed Mohamed Omar, Nuradin Abdullahi Sheikh Rashid, Ahmed Abdinasir Abdulle

**Affiliations:** 1 Faculty of Health Sciences and Tropical Medicine, Somali National University, Mogadishu, Somalia; 2 Center for Health Research and Innovation, Somali National University, Mogadishu, Somalia

**Keywords:** digital health, digital transformation, health systems strengthening, Somalia, universal health coverage

## Abstract

Universal Health Coverage (UHC) remains a major challenge in Somalia, where prolonged conflict, weak governance, fragmented service delivery, and donor dependence continue to undermine health system performance. Although digital health has emerged as a promising strategy for strengthening health systems, its implementation in Somalia has largely been characterized by fragmented, donor-driven, and disease-specific initiatives with limited integration into national health system reform. This Perspective argues that a major barrier to digital health-enabled progress toward UHC in Somalia is the fragmentation of governance, financing, health information systems, and implementation, rather than simply the absence of digital technologies. Drawing on recent evidence from Somalia and other low- and middle-income countries, we propose reframing digital health as strategic public infrastructure that integrates governance, service delivery, information systems, and financing to support health system transformation. We identify five strategic priorities: strengthening digital health governance, building interoperable digital architecture, accelerating digitally enabled primary health care, ensuring sustainable financing through effective public–private collaboration, and advancing digital equity with workforce capacity. Collectively, these priorities provide a policy-oriented framework for moving from fragmented digital initiatives toward a more integrated, resilient, and people-centred health system that could support progress toward Universal Health Coverage in Somalia.

## 1 Introduction

Universal health coverage is a core global health objective because it links access to essential services with financial protection, yet progress is slowest where conflict, displacement, weak institutions, and donor dependence undermine system-wide reform (1, 2). Somalia exemplifies this challenge: its UHC performance remains among the weakest reported, reflecting fragmented governance, shortages of health workers, inadequate medicines and infrastructure, and persistent inequities in access to care (3, 4). These constraints are compounded by heavy reliance on external assistance and out-of-pocket spending, while a large and heterogeneous private sector operates within limited regulatory and accountability structures (3, 5).

Digital health has emerged as an important enabler of health system strengthening because it can extend service reach, support workforce training and supervision, improve continuity of care, and strengthen data use in resource-constrained settings (6). In African settings, telemedicine, mobile health, and electronic records have shown substantial promise, but their impact depends on infrastructure, workforce capability, and integration with broader health information architecture rather than stand-alone deployment (7, 8). In Somalia, however, digital initiatives have largely evolved through pilots and donor-supported projects, including telemedicine efforts, in a context still marked by bandwidth limitations, absent national e-health standards, and fragmented reporting systems (9).

The policy concern in Somalia is therefore not simply the availability of individual digital technologies, but how digital health is governed and integrated within the wider health system. Somalia-specific evidence documents fragmented governance, donor dependence, weak regulatory capacity, parallel implementation arrangements, and limited integration of digital initiatives into national health-system reform (3, 10). Evidence from broader fragile and conflict-affected settings and other LMICs suggests that similar patterns, including unclear institutional roles, project-based implementation, weak interoperability, and parallel programmes, can undermine system integration and sustainability (2, 11). Accordingly, this Perspective considers fragmentation a major barrier to digital health-enabled progress toward UHC in Somalia, while using evidence from comparable settings to inform plausible mechanisms and policy options rather than treating such evidence as direct empirical evidence from Somalia. The next section examines how these documented challenges and proposed mechanisms relate to Somalia's digital health landscape.

To inform this Perspective, we drew on a targeted, non-systematic selection of recent peer-reviewed literature relevant to digital health, universal health coverage, health-system governance, interoperability, primary health care, financing, equity, and workforce capacity. Priority was given to studies directly examining Somalia, while evidence from fragile and conflict-affected settings, sub-Saharan Africa, and other LMICs was used where Somalia-specific evidence was limited and where it offered relevant comparative or conceptual insights. Sources were selected for their relevance to the policy questions and conceptual framework developed in this Perspective. Throughout the manuscript, Somalia-specific observations are distinguished from comparative evidence used to support proposed mechanisms and transferable policy lessons; the synthesis is therefore interpretive rather than a systematic or exhaustive review of the literature.

## 2 The fragmentation challenge: why current digital health efforts are not transforming the health system

Despite expanding global investment in digital health, Somalia's digital health landscape remains characterized by fragmented initiatives operating within a health system already constrained by financing limitations, infrastructure deficits, workforce shortages, and political and security instability (3, 9). Across sub-Saharan Africa, uncoordinated digital deployment has been associated with duplication, weak scalability, and poor interoperability, providing relevant comparative evidence for understanding similar risks in Somalia (11, 12). Somalia's broader UHC performance also remains weak. A 2025 Somalia-specific analysis, using a modified composite UHC index that combined essential service coverage and financial risk protection based on the 2020 Somali Health and Demographic Survey and the 2022 Somalia Integrated Household Budget Survey, reported a national score of 33.5 on a 0–100 scale (3). This study-derived measure provides evidence of low overall UHC performance but does not, by itself, establish digital fragmentation as its cause.

The challenge is therefore partly structural rather than solely technological. In Somalia, fragmented federal–state governance, limited regulatory capacity, and weak accountability mechanisms coexist with workforce maldistribution, limited rural coverage, and fragmented reporting systems (9). Somalia-specific evidence also documents substantial reliance on external health financing and parallel programmes, supply chains, and reporting arrangements involving humanitarian and development actors (3, 10). These arrangements may constrain national stewardship and coordination, although their specific effects on digital-health integration have not been established through causal analysis. Evidence from other LMICs indicates that fragmented mandates, weak enforcement, and project-based financing can contribute to disease-specific or donor-specific digital platforms, partial data flows, manual workarounds, and uneven compliance with common standards (11, 13). In the Somali context, these findings are therefore used as comparative evidence of plausible risks rather than as direct proof of causation.

We propose that these documented constraints may interact through several plausible pathways rather than constituting an empirically established causal sequence. Fragmented governance may hinder the development and enforcement of common standards; limited standardization may, in turn, contribute to non-interoperable platforms; and parallel reporting systems may increase reporting burdens and reduce the consistency and timely use of health information (11, 13). In Somalia, federal governance fragmentation, donor dependence, infrastructure limitations, and a large but weakly regulated private sector may further shape these pathways (3, 9). Evidence from Kenya and the wider African interoperability literature suggests that government stewardship, common standards, and multi-stakeholder coordination can support greater system integration (14, 15). although these experiences should be interpreted as comparative policy lessons rather than evidence that identical effects will occur in Somalia. Against this background, Somalia could benefit from moving from disconnected digital initiatives toward an integrated digital ecosystem embedded within broader health-system reform; the next section develops this as a proposed approach to health-system integration rather than an empirically established causal pathway.

## 3 Reframing digital health as a health system integrator rather than a standalone innovation

In this Perspective, digital health as public infrastructure refers to the shared institutional and technical foundations required for digital services to function across the health system rather than as isolated projects. These foundations include coordinated governance, common standards, interoperable information exchange, sustainable financing arrangements, and mechanisms that enable public, private, and humanitarian providers to participate within a common national framework. In the Somali context, this concept is distinct from an integrated digital health ecosystem: the public infrastructure provides the shared rules and foundations, whereas the digital ecosystem comprises the interconnected actors, platforms, services, and institutions that operate through them. Digital health functions as a health-system integrator when these shared foundations enable information, service delivery, financing, workforce functions, and accountability to connect across levels of care. This framing is particularly relevant in Somalia, where fragmented governance, donor dependence, weak interoperability, and a large but weakly regulated private sector currently constrain system-wide coordination (9, 11). The proposed mechanism is therefore institutional as well as technical. Governance can establish common rules, responsibilities, accountability arrangements, and standards; interoperability can enable information to move securely across providers and levels of care; sustainable financing can support shared infrastructure and reduce dependence on short-term, stand-alone projects; and integration into routine primary care and referral pathways can connect digital functions directly to service delivery. When these elements operate together, they could improve continuity of care, reduce duplication, strengthen the availability and use of health information, and support more coordinated allocation of resources. These relationships are proposed as a conceptual pathway for Somalia rather than as empirically demonstrated causal effects, and their effectiveness would remain dependent on workforce capability, infrastructure, institutional capacity, and equitable access (16, 17).

Within a WHO building-blocks perspective, digital transformation matters because it can reinforce several system functions simultaneously. Evidence from sub-Saharan Africa and other LMICs shows that digital systems can institutionalize real-time data use, improve patient management, strengthen performance monitoring, support supply and resource tracking, and enable more evidence-based allocation of funds and staff, but these gains depend on interoperability, long-term financing, and integration into routine primary care workflows (17). Interoperable architectures are especially important because fragmented records undermine both individual continuity of care and population-level decision-making; electronic records function as the connective tissue of a health system only when common standards, exchange protocols, and national stewardship are in place (16, 18).

This is why digital transformation should be framed as institutional reform, not technological modernization. Sustainable digital health in LMICs requires organizational change, government ownership, stakeholder coordination, monitoring, regulatory protection, and stable financing; without these, digitization tends to reproduce siloed programmes and existing inefficiencies rather than correct them (19). Fragile and conflict-affected settings further show that coordinated, context-tailored responses, community involvement, and governance-centred resilience are more decisive than technology alone in sustaining services during shocks (2, 20).

For Somalia, an integrated digital ecosystem could provide a shared architecture linking primary care, referrals, disease surveillance, workforce deployment, supply chains, contracting, and health financing. Such integration could reduce duplication, strengthen accountability between public and private actors, and support more transparent resource allocation, although these effects would depend on national standards, adaptive implementation capacity, sustainable financing, and equity safeguards for remote, displaced, and digitally excluded populations (21, 22). This framing positions digital health as a proposed component of wider health-system reform rather than as a standalone technological solution. The next section therefore identifies the strategic priorities that could operationalize this system-wide approach to digital health-enabled progress toward UHC.

## 4 Five strategic priorities for digital health–enabled universal health coverage

### 4.1 Priority 1. strengthen national digital health governance

Digital health governance in Somalia should be anchored in coordinated stewardship across the Federal Ministry of Health and the health authorities of the Federal Member States, with structured participation from private providers, humanitarian organizations, development partners, and other relevant national stakeholders (9, 23). A practical mechanism would be a formal federal–state digital health governance platform responsible for agreeing national standards, clarifying institutional responsibilities, coordinating partner-supported investments, and overseeing requirements for interoperability, privacy, cybersecurity, and data governance. Federal institutions would provide national policy direction and common standards, while Federal Member State health authorities would adapt and implement these standards within their service-delivery contexts and provide implementation feedback to the national level. Private and humanitarian providers should participate through agreed regulatory, reporting, and interoperability requirements rather than operating parallel digital arrangements. Progress could be assessed through indicators such as the establishment and regular functioning of a joint federal–state governance mechanism, the proportion of major digital health initiatives aligned with nationally agreed standards, adoption of common standards across Federal Member States, and compliance of participating public, private, and humanitarian platforms with agreed data-governance and interoperability requirements (9, 24).

### 4.2 Priority 2. build an interoperable national digital health architecture

Somalia should develop an interoperable national digital health architecture under the joint stewardship of federal and Federal Member State health authorities, with technical participation from public facilities, private providers, humanitarian organizations, development partners, and relevant digital-service actors. The principal implementation mechanism should be a nationally agreed interoperability framework that defines common data standards, exchange protocols, system interfaces, security requirements, and governance rules for connecting existing and future digital health platforms rather than replacing them with additional stand-alone systems (10, 21). Implementation could proceed incrementally by prioritizing high-value connections among health information systems, electronic records, surveillance platforms, referral services, and other essential health-system functions. Public, private, and humanitarian providers participating in the national health system should progressively align their digital platforms with these common standards and secure information-exchange requirements (9, 22). Progress could be monitored through indicators such as adoption of a national interoperability framework, the proportion of major digital health platforms conforming to agreed standards, the number or proportion of priority systems able to exchange data securely, and reductions in duplicate or parallel reporting arrangements. These indicators would assess progress toward interoperability without assuming that interoperability alone will produce improvements in UHC.

### 4.3 Priority 3. accelerate digitally enabled primary health care

Digitally enabled primary health care in Somalia should be implemented through coordinated action by federal and Federal Member State health authorities, primary care facilities, community health workers, private providers, humanitarian organizations, and development partners. The principal implementation mechanism should be the progressive integration of digital tools into existing PHC workflows rather than the creation of parallel digital programmes. This could include linking community-level platforms, facility records, referral systems, maternal and child health services, communicable- and non-communicable-disease programmes, and routine surveillance within an interoperable service architecture (17, 25). Evidence from LMICs and African settings suggests that integrated digital systems can support point-of-care decision-making, referral coordination, community health-worker activities, follow-up care, and more timely service and surveillance data, although these effects depend on interoperability, workforce capability, connectivity, and alignment with routine services (17, 25). In Somalia, digital PHC should therefore complement rather than replace frontline health workers and should be introduced alongside training, supervision, infrastructure support, and sustainable financing (19, 26). Progress could be assessed through indicators such as the proportion of PHC facilities and community health-worker programmes using interoperable digital tools, the proportion of referrals recorded and completed electronically, completeness and timeliness of routine PHC reporting, continuity of follow-up for priority services, and the proportion of frontline workers receiving relevant digital-health training. These indicators would measure implementation and service integration without assuming that digitalization alone will improve access, quality, or UHC outcomes.

### 4.4 Priority 4. create sustainable digital health financing and public–private collaboration

Somalia should move from short-term, project-based digital funding toward coordinated financing aligned with national digital health priorities, led by federal and Federal Member State health authorities in collaboration with development partners and private providers (10, 26). The principal mechanism should be pooled or coordinated investment tied to common interoperability, accountability, and data-governance requirements, while public–private partnerships should support infrastructure and service delivery within nationally agreed standards (14, 15). Progress could be assessed through the proportion of digital investments aligned with national priorities, the share of major partner-funded projects using common standards, and the number of formal public–private arrangements operating under agreed governance requirements.

### 4.5 Priority 5. ensure digital equity and workforce readiness

Digital health expansion in Somalia should be accompanied by explicit equity and workforce safeguards, led by federal and Federal Member State health authorities in collaboration with training institutions, providers, and development partners. Implementation should integrate accessibility, affordability, connectivity, and digital-literacy considerations into programme design, while embedding digital competencies, supervision, and continuing professional development within the health workforce (6, 19). Particular attention is needed for rural populations, displaced communities, women, persons with disabilities, and other groups at risk of digital exclusion. Progress could be assessed through workforce training coverage, geographic and population-level access to digital services, accessibility of digital platforms, and identified gaps in connectivity or use. These measures could help reduce the risk that digital transformation reproduces existing inequities (22, 24).

## 5 From technology adoption to system transformation

The five strategic priorities are interdependent rather than separate reform tracks. Governance provides the institutional authority for establishing and enforcing common standards, while interoperability depends on those standards being adopted across public, private, and humanitarian systems. Financing is similarly linked to governance: without alignment to national priorities and common interoperability requirements, new investments may reproduce parallel platforms and fragmented reporting arrangements rather than strengthen system integration (11, 19). Coordinated governance, financing, and interoperability therefore provide mutually reinforcing conditions for digital health to become embedded within wider health-system reform rather than remain a collection of stand-alone technologies (16, 19).

Digitally enabled primary health care likewise depends on workforce readiness, connectivity, and equitable access. Interoperable systems may have limited practical value if frontline workers lack the capacity to use them effectively or if rural, displaced, poor, or otherwise digitally excluded populations cannot access the services they support (17, 22). Public–private collaboration cuts across these priorities because private and humanitarian actors need to participate within common governance, financing, and information-exchange arrangements rather than parallel systems (15, 27). Taken together, these interactions constitute a proposed system-transformation pathway for Somalia rather than a linear or empirically established causal sequence.

Figure 1 summarizes this proposed conceptual pathway, illustrating how interactions among the five strategic priorities could support greater health-system integration and progress toward Universal Health Coverage in Somalia.

**Figure 1:**
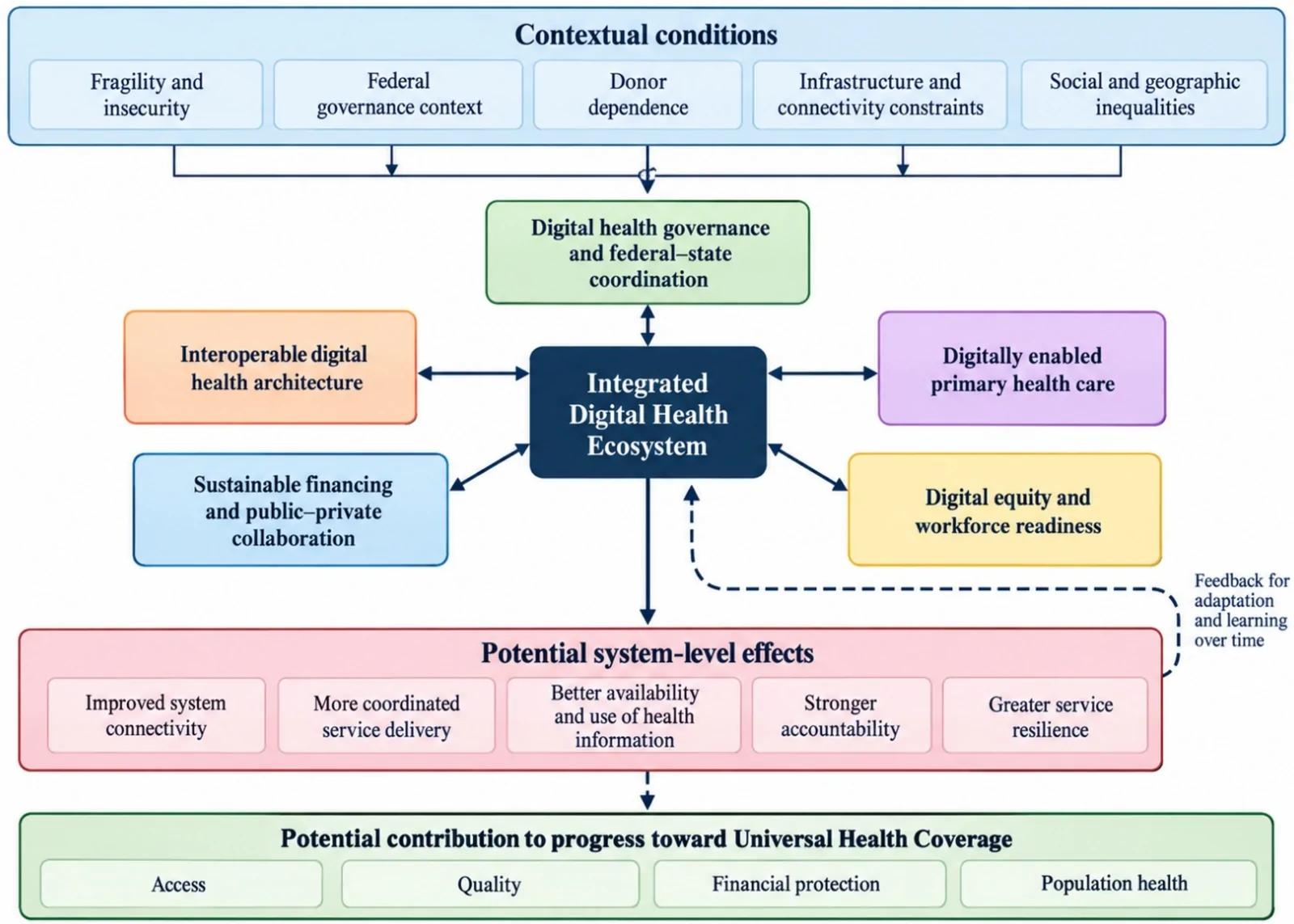
Proposed conceptual pathway for digital health-enabled health-system integration in Somalia. The framework shows five interacting strategic priorities: digital health governance and federal–state coordination, interoperable digital health architecture, digitally enabled primary health care, sustainable financing and public–private collaboration, and digital equity and workforce readiness within an integrated digital health ecosystem. Together, these interacting priorities may support improved connectivity, more coordinated service delivery, better use of health information, stronger accountability, and greater resilience, which could contribute to progress toward Universal Health Coverage. The pathway is conceptual rather than empirically established and is shaped by contextual conditions such as fragility, donor dependence, infrastructure constraints, federal governance arrangements, and social and geographic inequalities.

## 6 Conclusion

Somalia's progress toward Universal Health Coverage could be supported by moving from fragmented digital initiatives toward a more integrated and nationally coordinated digital health ecosystem embedded within wider health-system reform. The evidence considered in this Perspective highlights governance fragmentation, donor dependence, limited interoperability, financing constraints, workforce readiness, and inequitable access as important challenges, while evidence from comparable LMIC and fragile settings provides relevant lessons for possible policy responses.

The proposed framework therefore positions digital health as shared public infrastructure supported by coordinated governance, interoperable architecture, digitally enabled primary health care, sustainable financing and public–private collaboration, and digital equity with workforce readiness. These priorities should be understood as an interconnected policy pathway rather than as an empirically established causal sequence. If adapted to Somalia's federal, institutional, and service-delivery context, their coordinated implementation could support stronger health-system integration, resilience, accountability, and progress toward UHC. Further policy implementation and empirical evaluation will be needed to determine how these proposed mechanisms operate in practice.

## Data Availability

The original contributions presented in the study are included in the article/Supplementary Material, further inquiries can be directed to the corresponding author.
